# Vascularization of kidney organoids: different strategies and perspectives

**DOI:** 10.3389/fruro.2024.1355042

**Published:** 2024-05-21

**Authors:** Irina Raykhel, Masaki Nishikawa, Yasuyuki Sakai, Seppo J. Vainio, Ilya Skovorodkin

**Affiliations:** ^1^ Developmental Biology Laboratory, Disease Networks Research Unit, Faculty of Biochemistry and Molecular Medicine, University of Oulu, Oulu, Finland; ^2^ Laboratory of Organs and Biosystems Engineering, Department of Chemical System Engineering, University of Tokyo, Tokyo, Japan; ^3^ Infotech Oulu, University of Oulu, Oulu, Finland; ^4^ Kvantum Institute, University of Oulu, Oulu, Finland

**Keywords:** kidney organoids, vascularization, organ-on-a-chip model, *in vitro* vasculature, biomedicine, microfluidistics, nephrogenesis

## Abstract

Kidney diseases such as glomerulopathy and nephron dysfunction are estimated to grow to more than 900 million cases by 2030, in 45% of which kidney transplantation will be required, representing a major challenge for biomedicine. A wealth of progress has been made to model human diseases using induced pluripotent stem cells (iPSCs) *in vitro* differentiated to a variety of organoids, including kidney organoids, and in developing various microfluidics-based organ-on-a-chip (OoC) systems based on them. With the combination of targeted gene editing capacities, relevant polymorphic genetic variants can be established in such organoid models to advance evidence-based medicine. However, the major drawback of the current organoid disease models is the lack of functional endothelial vasculature, which especially concerns the kidney, the function of which is strongly associated with blood flow. The design of novel medical devices using tissue engineering approaches such as kidney organoids is also strongly dependent on the understanding of the fundamental principles of nephrogenesis and the vascularization of organs and tissues. Developmental vascularization of the kidney has been an area of intense research for decades. However, there is still no consensus among researchers on how exactly the vascularization of the kidney occurs in normal and pathological conditions. This lack of consensus is partly due to the lack of an appropriate model system to study renal vascularization during nephrogenesis. In this review, we will describe recent progress in the areas of kidney vasculature development, kidney organoids in general and assembled on microfluidic devices in particular. We will focus on the *in vitro* vasculature of kidney organoids in microfluidic OoC model systems to study kidney diseases and on the perspectives of tissue engineering for the modeling of kidney diseases and the design of bioartificial medical devices. We also aim to summarize the information related to the key mechanisms of intercellular communication during nephrogenesis and the formation of the renal vasculature in an OoC setup.

## 1 Introduction

Chronic kidney disease is one of the most common causes of death and one of the leading kidney diseases. Chronic kidney disease affects approximately 10% of the population worldwide according to statistics from the American National Kidney Foundation (1). More than 40,000 of kidney transplants are performed every year, the need for kidney transplants is very high, and there are still people who die without the opportunity to receive a life-saving transplant (2). Renal replacement therapy is a complicated and expensive medical procedure. The deficiency of transplant kidneys and the morbidity associated with dialysis have prompted medical biologists to develop novel technologies for the treatment of kidney dysfunctions. While congenital malformations and epigenetic factors cause end-stage kidney disease, little is understood regarding the cell biology underlying these disorders. New diagnostic methods and treatments therefore need to be developed. Cutting-edge studies in genomics, developmental biology, pluripotent stem cells (PSCs), and tissue engineering are needed to address these scientific and translational challenges.

The development of advanced model systems aimed at studying disease mechanisms and elaborating new therapies is crucial. These model systems should closely match the development and function of both healthy and diseased human kidney. Renal organoids are generally hoped to be the appropriate model system to study the development of healthy and diseased kidney. However, the main function of the kidney is blood filtration, and until now, no organoids or other corresponding kidney models have been proposed to maintain this function.

The functional units of the kidney nephrons are assembled through a process called nephrogenesis. In humans, this process stops around the 36th week of pregnancy (3). According to many, but not all, reports, the adult kidney lacks a population of nephron progenitors and is unable to generate new nephrons or regenerate existing ones (4). Deep understanding of kidney development might lead to the discovery of progenitor cell populations that could be utilized in regenerative medicine to repair damaged kidney tissue (4).

One of the major recent breakthroughs in the field of biomedicine is the application of organoids differentiated from stem cells, which opened new opportunities for studying the basic principles of organ development, creating disease models, and using them in the discovery of new medical technologies. The application of patient-derived stem cells has provided new perspectives for personalized medicine, and perhaps even in personalized tissue and organ engineering. Already, there are several publications demonstrating the great potential of renal organoids generated from patient-specific cells for the selection of the best drugs or methods for the treatment of kidney diseases (5–14) for certain patients. However, the renal organoids generated using these approaches have reproduced kidneys at the developmental rather than the adult stage. Specifically, the lack of vascularization does not allow generating an *in vitro* model of a functional kidney that reproduces the morphology and physiology of an adult kidney. Thus, *in vitro* vascularization of renal organoids is an extremely important challenge in the development of a clinically relevant *in vitro* model system aimed at treating renal dysfunctions and developing novel therapies for kidney diseases.

In this paper, we review the progress made in the vascularization of kidney organoids. We discuss the role of endothelial cells (ECs) during kidney organoid development and analyze the various strategies in the vascularization of kidney organoids, the source of ECs used for these purposes, and the challenges in the generation of a functional vascularized kidney organoid *in vitro*.

## 2 Kidney organoids

Currently, there are many different protocols for generating human- or animal-derived organoids. Thus, it is extremely important to evaluate how well the multicellular complex resulting in each particular case meets the definition of an organoid. According to the modern definition, an organoid is an artificial self-organizing tissue that shows resemblance to an organ and satisfies the following criteria: 1) recapitulates the developmental program to result in proper identity (PSC-derived organoid); 2) retains the identity of the organ from which the cells were isolated (adult tissue-derived organoid); 3) contains more than one cell type, as in the organ; 4) exhibits at least one specialized function of the organ; and 5) develops and matures according to intrinsic organizing principles, as in the organ (15–17). Thus, it is currently possible to define the kidney organoid as a heterocellular structure grown *in vitro* and reproducing at least the nephrons, but so far without the function of glomerular filtration and perfusion of the filtrate into nephron tubules (18). PSCs, combining pluripotent embryonic stem cells (ESCs) and induced pluripotent stem cells (iPSCs) together with adult stem cells, are the source of cells from which organoids can be differentiated. To date, organoids have been obtained for many organs (17). Work on the development of kidney organoids is being carried out very intensively and is already showing encouraging results, although many obstacles still need to be overcome for the translation of the corresponding laboratory research into medical technologies.

### 2.1 *In vitro* differentiation of PSCs to assemble kidney organoids

The mammalian metanephric kidney is a highly complex organ responsible for many functions. The main ones include filtering the blood from waste products, maintaining the electrolyte and pH balance of the body fluids, regulating the bone mineral metabolism, and controlling the blood pressure and blood composition. Many of these functions are performed by the nephron, which is a main functional unit of the kidney.

Therefore, the main goal of the differentiation protocols is to obtain the structure that recapitulates many of the key structural and functional features of the nephron. Accordingly, so much effort has been put into studying in detail all the stages of nephron development.

The first protocols for differentiating kidney organoids from PSCs have been established (6, 9, 19–32) (**Table 1**). From the outset, the protocols were based on the principle of following the natural processes of embryonic kidney differentiation. Hence, it is necessary to study all of the steps of kidney development and to learn how to repeat these steps sequentially.

**Table 1:** Main protocols for human pluripotent stem cell (hPSC)-derived kidney organoids and renal tissue differentiation.

Differentiation protocol	Metanephric mesenchyme	Ureteric bud	Endothelial cells	Main protocol characteristics	Reference
Taguchi et al., 2014	Yes	No	ECs not detected	Identification of the MM precursors, different from the UB precursors, made it possible to form a kidney organoid, including glomeruli with podocytes and renal tubules with proximal and distal sections and a clear lumen.	(19)
Takasato et al., 2014	Yes	Yes	ECs not detected	The protocol of the differentiation of hESCs results in the synchronous induction of UB and MM that forms a self-organizing structure with nephron formation.	(20)
Freedman et al., 2015	Yes	No	ECs were detected	A protocol was developed to obtain nephron-like kidney organoids containing cell populations with characteristics of proximal tubules, podocytes, and endothelium.	(6)
Morizane et al., 2015	Yes	No	ECs were detected	The high induction efficiency of nephron progenitors allowed developing a protocol for the generation of nephron-like kidney organoids containing epithelial nephron-like structures expressing markers of podocytes, proximal tubules, loops of Henle, and distal tubules.	(9)
Takasato et al., 2015	Yes	UB-like cells	ECs were detected	The knowledge that collecting ducts and nephrons have different spatiotemporal origins has allowed the creation of kidney organoids containing nephron progenitor-derived podocytes, Bowman’s capsules, and renal tubules, as well as UB-like cells, stromal cells, and endothelial cells.	(21)
Taguchi et al., 2017	No	Yes		By studying the different origins and developmental processes of UB and nephron progenitors, a differential induction protocol for each lineage was established. The kidney organoid was formed from UB combined with NP and SP and had higher-order renal structures including glomeruli and renal tubules located at the tips of the collecting ducts.	(24)
Mae et al., 2018	No	Yes		The protocol allowed the generation of UBs from the AIM with branching UB tissues and WD cells.	(25)
Przepiorski et al., 2018	Yes	No	ECs were detected	A relatively simple and cost-effective method for the large-scale production of kidney organoids with nephrons containing podocytes, proximal and distal tubule segments, presumptive collecting ducts, endothelial cells, and interstitial cells.	(26)
Garreta et al., 2019	Yes	No	ECs were detected	The protocol produced kidney organoids with multiple nephron-like structures that were segmented into typical nephron components, including proximal tubules.	(27)
Low et al., 2019	Yes		ECs organize the vasculature network	This protocol generates kidney organoids with segmentally patterned nephron structures, and glomerular podocytes producing appropriate levels of *VEGFA* define the resident vasculature.	(28)
Uchimura et al., 2020	Yes	Yes	ECs not detected	The independent generation of two populations of renal progenitor cells—MM cells and UB-like cells—yielded kidney organoids with a collecting system.	(29)
Tan et al., 2020		Yes	ECs form a vascular network	The protocol allowed the differentiation of mESCs to be directed into UB progenitor cells. Later, the co-culture of mESC-derived UB cells with pMM induced nephrogenesis. In the resulting kidney organoids, the pMMs developed nephron structures, and the mESC-derived UB cells formed numerous collecting ducts connected with nephron tubules.	(30)
Howden et al., 2021		Yes		The protocol focused on the production of ureteric epithelium by inducing the distal nephron epithelium for subsequent use in collecting duct formation.	(31)
Shi et al., 2023		Yes		Protocol for the differentiation of UB organoids into functional collecting duct organoids.	(32)

ECs, endothelial cells; MM, metanephric mesenchyme; UB, ureteric bud; hESCs, human embryonic stem cells; NP, nephron progenitors; SP, stromal progenitors; AIM, anterior intermediate mesoderm; WD, Wolffian duct; mESCs, mouse embryonic stem cells; pMM, primary metanephric mesenchyme.

#### 2.1.1 Development of the mammalian kidney

**Figure 1:**
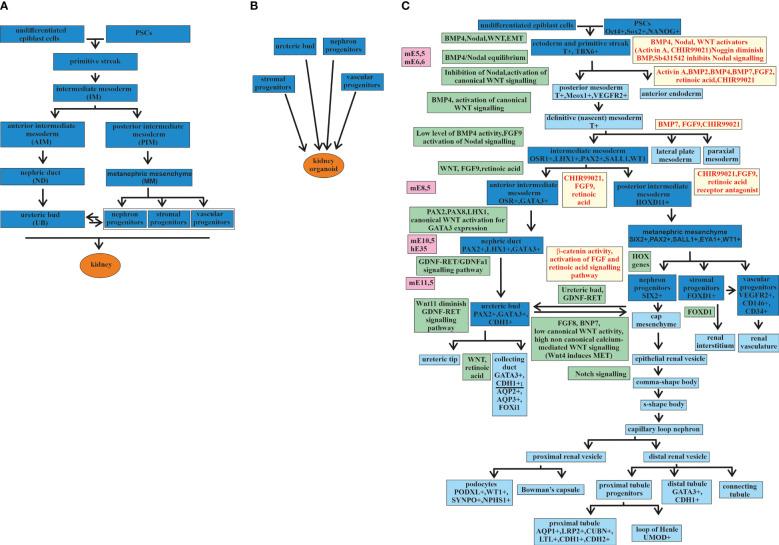
Major stages of kidney development and kidney organoids. **(A)** Major stages of kidney development from an undifferentiated epiblast. **(B)** Four major progenitors [i.e., ureteric bud (*UB*), nephron progenitors (*NP*), stromal progenitors (*SP*), and vascular progenitor (*VP*)] are required for the formation of a kidney organoid. **(C)** Major stages of kidney development (the main stages marked in *deep blue* and the additional stages in *blue*) from undifferentiated epiblast cells and kidney organoids from pluripotent stem cells (PSCs), with signaling pathways and transcription factors discovered during embryonic development (marked in *green*) and used in directed differentiation protocols (marked in *yellow*). *mE*, mouse embryonic day; *hE*, human embryonic day.

The kidney develops from the mesoderm germ layer, one of three germ layers—the endoderm, the mesoderm, and the ectoderm—formed from the primitive streak (33) (**Figure 1**). Differentiation from the primitive streak leads to the formation of the intermediate mesoderm (IM). One precursor of the embryonic kidney, the ureteric bud (UB), differentiates from the IM in an independent direction from three other precursors: the nephron progenitors (NPs), the stromal progenitors (SPs), and the vascular progenitors (VPs) (**Figure 1A**). The separation of directions occurs at the stage when the IM is differentiated into the anterior IM (AIM) and the posterior IM (PIM). From the AIM, the nephric duct (ND) is formed, which gives rise to the UB. The PIM differentiates into the metanephric mesenchyme (MM), from which the NPs, SPs, and VPs are formed (33–37). All four precursors are required for kidney development or renal organoid formation (**Figure 1B**) (36).

Thereafter, the stages of kidney development are presented in more detail, taking into account the expressing factors that are involved in this process in order to determine the stages of the differentiation of PSCs into a kidney organoid (**Figure 1C**).

Mouse embryo studies have determined that, at stage E5.5, the anterior visceral endoderm expresses *Lefty*, *Cer­1*, and *Dkk­1*. These are inhibitory factors that counteract the Nodal, bone morphogenetic protein (BMP), and Wnt signaling pathways. The primitive streak is formed on the opposite side, where the Nodal, BMP, and Wnt signaling pathways remain active. The activation of Wnt3 stabilizes nuclear β­catenin in the primitive streak in response to the expression of BMP4 in the ectoderm layer. These cause the transcriptional activation of primitive streak genes such as *nodal*, *T* (also known as Brachyury), *mixl1*, and *Eomes*. Thus, formation of the primitive streak from the posterior epiblast is induced by the crosstalk between Nodal, BMP4, and the canonical Wnt pathway (33). Mesoderm cells are derived from the posterior side of the primitive streak. After exiting the primitive streak, these cells express the following mesoderm markers: *T*, *Meox1*, and *Kdr* (other names are *Flk1* or *VEGFR2*) (33, 38–40). The opposite anterior side of the primitive streak forms endodermal tissues with the expression markers *Mixl1*, *Sox17*, *Foxa2*, and *Eomes* (33, 41). Depending on the gradient of Nodal and BMP4 expression along the anteroposterior axis of the primitive streak, either the mesoderm or the endoderm is induced. In the case of a high level of BMP and a low level of Nodal expression in the primitive streak, the mesoderm is induced. A low level of BMP and a high level of Nodal expression in the primitive streak activate the induction of the endoderm (33, 42, 43).

It has been known from the study of mouse embryo development that the trunk mesoderm develops from the posterior primitive streak. The trunk mesoderm is the progenitor of the paraxial mesoderm, the lateral plate mesoderm, and the IM. The postnatal mammalian kidney has the mesoderm originated and formed from the IM expressing the transcriptional regulator *Osr1* (44, 45). The main steps of kidney development as a result of the interactions among the different *Osr1* ^+^ derivatives were established by a study on mouse kidney using organ culture experiments (46). Synergistic regulation by several morphogens, including BMP4, Nodal, and FGF9, provides the differentiation of the IM from the trunk mesoderm (47–50). The subsequent IM regionalization is determined by the distribution of the morphogenetic signals along the anterior–posterior axis and gives rise to three structures: the pronephros, the mesonephros, and the metanephros (44). Kidneys generate from the metanephros (44).

The IM gives rise to two directions of development. One goes through the AIM directed by Wnt, FGF9, and retinoic acid. The AIM by *PAX2*, *PAX8*, *LHX1*, and canonical Wnt activation for *GATA3* expression produces the ND. The ND then differentiates to the UB through GDNF-RET/GDNFR alpha-1 signaling (51–53). The epithelial UB interacts with the MM cells, initiating the process of branching morphogenesis (54–56). During this process, the UB tips and the MM cells exchange signals, leading to multiple repetitions of UB stalk extensions and another round of interaction of the UB tips with the MM cells, continuing UB branching (57–60). This leads to the formation of a kidney collecting duct (CD) system (61).

Recent anatomic and molecular characterization of the human nephron development has confirmed the presence of *GATA3* ^+^ and *CDH1* ^+^ in the epithelium of the UB-derived CD and the distal tubule determining a connecting segment between the CD and the distal tubule (24, 62–64). This correlates well with the previously proposed model, according to which the nascent CD epithelium and the nephron precursor cells possess distal identity. This causes the formation of an interconnection of the lumen of the CD and the nephron-derived epithelial tubules (65–67). The expression markers *AQP2*+, *AQP3*+, and *FOXi1*, among others, more accurately identify the part of the kidney CD (32, 63, 64). The co-expression of many genes in the CD and the distal tubule reflects the complex interaction between them during development, which requires further research.

The second direction of the development of the IM is through the PIM. The PIM develops into the MM, which gives rise to the NPs, SPs, and VPs. The crosstalk between the MM cells and the UB tips, which are involved in branching morphogenesis and the formation of the CD system, is also required for the formation of the cap mesenchyme, which contains the progenitor cells of the entire nephron (49). The MM differentiates into three directions, establishing three populations with diverse expression profiles (50). The first population with *Six2* ^+^ *Cited1* ^+^ MM cells forms the renal vesicle, from which later originated all components of the main body of the nephron, such as podocytes and the connecting segment plumbing into the ureteric epithelium. The second population with *Foxd1* ^+^ MM cells forms pericytes and mesangial cells (44). The third population with *Flk1* ^+^ (*Kdr*/*Vegfr2*) MM cells forms vascular progenitor cells, which later will participate in vasculogenesis together with additional angiogenic external support in the vascularization of the glomerulus (44).

Thus, the structures formed along two different directions of development, i.e., the UB on the one hand and the NPs, SPs, and VPs on the other hand, at a certain stage, begin to interact with each other and thus continue to develop into structures from which the adult kidney is obtained. The main steps of kidney development are presented in **Figure 1**.

#### 2.1.2 The kidney organoid protocol mimics the natural process of kidney development

Understanding the general principles and mechanisms of kidney development has aided the search for options to recapitulate the morphology of the organ *in vitro* as an attempt to mimic the natural developmental process. The first successful assays to establish the protocol for the differentiation of PSCs to three-dimensional (3D) kidney organoids were based on detailed knowledge of mouse embryonic kidney development (6, 9, 19, 22, 68). The protocols were based on the induction of PSCs in different combinations by BMP, Nodal, Wnt, and activators of the canonical Wnt signaling pathway, which leads to the formation of the primitive streak. The induction of ESCs was performed by the activation of the canonical Wnt pathway, which leads to the expression of a stabilized form of β-catenin and the differentiation of monolayered human ESCs (hESCs) into primitive streak cells that express the *T*, *MIXL1*, and GSC expression markers. Enhancement of BMP signaling using SB­431542, an inhibitor of the Nodal signaling pathway, and activation of canonical Wnt signaling promote the induction of the posterior primitive streak and develop the mesoderm. Various modes of the formation of 3D embryoid bodies, within which cell–cell interactions occur, enhance the BMP-mediated activation of Nodal signaling and lead to the formation of the primitive streak. The presence of the GSK3 inhibitor (CHIR99021) activates the canonical Wnt signaling pathway and facilitates the differentiation process (33).

Induction of the formation of the primitive streak in the PSC differentiation protocol is realized by activin A and CHIR99021, or activin A, BMP2, BMP4, BMP7, FGF2, and retinoic acid in order to reproduce the natural process. The induction of the IM is activated using BMP7 and CHIR99021, or FGF2 together with retinoic acid. Some protocols assume that hESC­derived posterior primitive streak cells can spontaneously form a lateral plate mesoderm expressing the *OSR1* and *FOXF1* expression markers under growth factor-free medium conditions. Later addition of FGF9 results in the formation of an IM expressing the *OSR1* ^+^ *LHX1* ^+^ *PAX2* ^+^ expression markers (20). In the next step, long incubation with CHIR99021, FGF9, and the retinoic acid receptor antagonist leads to the development of the posterior IM and MM. On the other hand, short incubation with CHIR99021, FGF9, and retinoic acid leads to the development of the AIM and, later, to the formation of the ND and UD. Lineage tracing and genetic knockout of NP lineage-specific genetic studies identified spatiotemporally distinct origins of the MM (NP+SP) and UB (19, 69). More detailed study of the AIM or PIM specification steps led to the design of a sufficient differentiation protocol of inducing the UB from PSCs (24). The strategy to differentiate separately and later integrate the NP- and UB-derived components in one organoid produced structures that more closely resemble mammalian kidneys (24, 29, 31, 70).

#### 2.1.3 Further improvement of the protocols for the generation of kidney organoids

The induction of mouse ESC-generated MM and UB separately and their subsequent combination resulted in the formation of a UB-derived collecting system with branches in the correct manner (33). Thus, the idea of recapitulating kidney development was realized. This separate induction and the subsequent reassembly strategy showed that reaggregated organoids developed the architectures of the kidney organoid similar to that of the embryonic kidney. This includes the peripheral progenitor niche and the internally differentiated nephrons, which are interconnected by the branched ureteral epithelium (33).

The finding that the AIM differentiates from the anterior primitive streak formed the basis of another assay aimed at establishing a reliable method for the induction of the development of the branching UB tissue from human pluripotent stem cells (hPSCs). This method enabled an efficient differentiation of hPSCs into Wolffian duct cells (25).

A continuation of the idea to induce different cells that constitute embryonic kidneys separately and then reassemble them together was the protocol suggested by Tsujimoto et al. (70). They individually generated metanephric NP, mesonephric NP, and UB cells from hPSCs. In this culture system, these paraxial, lateral plate mesoderm and mesoderm-like cells were mixed together and subsequently differentiated into glomeruli, renal tubules, and CDs *in vitro*. The resulting kidney organoid was then vascularized *in vivo* by transplantation under the kidney capsule of immunodeficient mice (70). This was a successful trial to create a model system recapitulating the nephrogenic interactions between metanephric NPs and UB cells *in vitro*.

The processes of optimizing the previous and of creating new protocols continue. Attempts are being made to overcome the various limitations that the previous protocols had, such as the low diversity of the kidney cell types and their insufficient maturity and yield.

Identification of the previously undetected compartments within the organoids was accomplished using high-content image analysis and single-cell RNA sequencing (RNA-seq) (7). Both methods have been used to identify conditions that significantly expand the vascular endothelium.

Differentiation of mature post-mitotic podocytes from hPSCs was reported in Musah et al. (71) solely using defined chemical factors. No subpopulation selection or genetic manipulation of hPSCs was applied in the protocol. The authors reported high efficiency (>90%) of their method. They developed a feeder- and serum-free protocol to efficiently obtain glomerular podocytes from both hESCs and hPSCs, resulting in cells with morphological, molecular, and functional characteristics of mature kidney glomerular podocytes. Among them, the protein expression of nephrin, podocin, and WT1 was shown. Expression of the podocyte lineage specification genes *SYNPO*, *PODXL*, *MAF*, and *EFNB2* was also shown, with a corresponding decrease in the expression of the NP genes *PAX2*, *SALL1*, and *EYA1* and the pluripotency genes *POU5F1*, *SOX2*, *MYC*, and *NANOG*. Global transcriptomic analysis of hPSC-derived podocytes complements these characterizations. The strategy of this new protocol was based on the identification of specific extracellular matrix (ECM) proteins, such as laminin-511 and the laminin-511 E8 fragment, which specifically support the adhesion of hPSCs and their differentiated derivatives through the high expression of the β1 and αvβ5 integrins on their cell surface. The novel combination of differentiated factors in the medium, consisting of BMP7, activin A, vascular endothelial growth factor (VEGF), retinoic acid, and CHIR99021, directs the differentiation into mature, terminally differentiated podocytes. Another novelty of this protocol is that fibroblast growth factors such as FGF2 or FGF9 do not need to be added to induce terminally differentiated podocytes. The high levels of differentiation and functionality was shown by the ability to form within a microfluidic organ-on-a-chip culture device to build a human kidney glomerulus chip that mimics the structure and function of the kidney glomerular capillary wall.

The robustness, transferability, and reproducibility of the protocol for the differentiation of kidney organoids are critically important parameters for the further application of hPSC-derived kidney organoids in personalized medicine and functional genomics. Analysis of the sources of transcriptional variation showed significant variations between experimental batches, particularly in genes associated with temporal maturation (72). Single-cell analysis revealed a shift in the nephron patterns and the proportions of component cells.

One of the important tasks is to reduce the generation of off-target cells, which still remains a big challenge.

In Subramanian et al. (73), several human induced PSC (hiPSC) lines were profiled to show how organoid composition and development are comparable to human fetal and adult kidneys. Variability in the cell proportions was found between the different iPSC lines, mainly due to off-target cells. It is very interesting to note that the analysis of organoids transplanted under the mouse kidney capsule showed a decrease in the number of off-target cells, which might indicate a very important role for the functional vascularization of kidney organoids.

The comparison of directed differentiation protocols by single-cell transcriptomics with single-cell transcriptomes of fetal and adult kidney cells detected that protocols generate a diverse range of kidney cells with differing ratios, that the organoid-derived cell types are immature, and that 10%–20% of cells are non-renal (74). The analysis allowed identifying ligands, receptors, and transcription factor networks associated with fate decisions. Inhibiting the corresponding pathways improved organoid formation without affecting kidney differentiation, highlighting the power of single-cell technologies to characterize and improve organoid differentiation.

In another study, the authors independently differentiated two kidney progenitor cell populations, i.e., MM and UB-like cells, in order to generate human kidney organoids with improved maturation of the collecting system (29). The hormones aldosterone and arginine vasopressin were identified as critical to promoting the differentiation of CD cell types, including both principal and intercalated cells. This paper also showed that Notch signaling regulates the ratio of principal cells to intercalated cells during organoid development.

The authors established the protocol of a two-dimensional (2D) method of differentiating hPSCs into Wolffian duct cells and the procedure to generate 3D Wolffian duct epithelial structures that can form branching UB tissues (25). Subsequently, a method to generate UB organoids with epithelial polarity, tubular lumen, and repeat branching morphogenesis was developed (25).

Recently, a 3D branching UB organoid culture model derived from either primary UB progenitor cells or hPSCs has been established. This organoid model mimics the kidney branching morphogenesis *in vitro* and allowed obtaining the mature CD system from immature UB progenitors (75).

In addition, a new method for the *in vitro* differentiation of SPs from mouse PSCs was reported. To generate renal organoids, the SPs were assembled with two differentially induced parenchymal cell populations, the NPs and UBs. The resulting organoid showed improved UB branching and appropriate localization of NPs at the tips of UBs (76).

ESCs were used in the method of dissociation–reaggregation to form organotypic kidney structures (77). This method was later refined with the ability to manipulate different types and populations of embryonic kidney cells in order to monitor their influence on organoid development (78).

The analysis of iPSC-derived kidney organoids revealed that the resulting renal structures are surrounded by progenitor (nascent) vascular ECs, which, however, are not capable of forming a proper vasculature pattern (6, 21).

The use of several protocols produced PSC-derived organoids containing a population of ECs that were not organized into a vasculature and did not invade the glomeruli (6, 21, 79). Importantly, thus far, no protocol has generated a kidney organoid with a functional vasculature.

To increase the population of ECs in the resulting organoid, VEGF was added during organoid differentiation in the study by Czerniecki et al. (7). An approximately 10-fold increase in the number of ECs expressing CD31 and VE-cadherin did not compromise organoid formation. However, this also did not lead to appropriate interactions between the ECs and podocytes to form the glomerular basement membrane (GBM).

The next optimization along this path was to create dynamic modulation of Wnt signaling to control the relative proportions of proximal *versus* distal nephron segments, thereby creating a correlative level of *VEGFA* to determine the resident vasculature (28). Single-cell transcriptomic analysis of the EC populations determined a subpopulation of NP cells that contributed to the resident vasculature. The ECs of this population have already established a gene regulatory network responsible for defining the endothelial subtypes. The different distributions of the set of markers—the endothelial progenitor cell markers *KDR* and *FLT1* (*VEGFR2* and *VEGFR1*); the mature endothelial marker *PECAM1*/*CD31*; the NP cell marker *SIX1*; the arterial markers *NOTCH4*, *DLL4*, and *CXCR4*; and the venous markers *NR2F2* and *EPHB4*—provided the first evidence of the existence of specific endothelial subtypes in the vascular network within hiPSC-derived kidney organoids. It is extremely important to emphasize that this differentiation on the different subtypes occurred even in the absence of exogenous vascular growth factors (7) or sheer stress (80).

### 2.2 Kidney organoids on microfluidic chip

Considering the immensely complex organization of the mammalian kidney, reproducing its entire morphology and function *in vitro* would be an extremely challenging task. Therefore, many projects have focused on the recapitulation *in vitro* of at least some morphological or functional components of the kidney, e.g., a model of glomerular filtration or the renal tubule representing the re-absorption function, among others. An elegant and logical solution was to build these miniaturized renal “units” on a microfluidic platform.

The use of microfluidic chambers or so-called microfluidic chips, also known by other names such as “lab-on-a-chip,” became widely used in biomedical research about 20 years ago (81). It relies on the use of designs that can process small quantities of liquids using the channels that have microscale dimensions, i.e., typically tens to hundreds of micrometers. The strength of this method is that the microfluidic chambers (or microfluidic chips) can be used to create complex, controlled microenvironments in microchannels. The microfluidic method can provide a sophisticated cell bioanalysis platform by integrating multiple steps including fluid control, cell culture, cell capture, cell–cell and cell–matrix interactions, cell lysis, cell signaling, and biochemical detection in a single device. When research is carried out to study a whole organ or organoid, this method is called organ-on-a-chip (OoC) (78). The main goal of the OoC method is not to recreate an entire living organ with all its functions. The reconstruction of at least one critical function for each model should be considered a great success.

One method for the development of microfluidic systems is photolithography using transparent and gas-permeable polydimethylsiloxane (PDMS) (82). Another widely used method is 3D printing (83). A large list of commercially available microfluidic cameras is also available (82).

Currently, a wide variety of microfluidic devices are used to study the biological processes taking place in the vascular, respiratory, nervous, digestive, and excretory systems (84).

Several studies have aimed to reproduce the renal proximal tubular system *in vivo* using microfluidic devices (85–88). A bioprinting method to create 3D human proximal tubules *in vitro*, which are inserted into a perfused tissue microfluidic chip, has been reported (89, 90). In these studies, a 3D kidney tissue allows the proximal tubular epithelium and the vascular endothelium to be co-cultured, making this 3D vascularized proximal tubule model suitable for studying renal reabsorption and the crosstalk between the epithelium and the endothelium. Another type of kidney-on-a-chip platform, which is based on a PDMS mold, has been reported to allow the reconstitution of the human kidney vascular tubule unit *in vitro* using patient-derived cells (91). The subsequent variant of the microfluidic platform for the co-culture of the epithelium and endothelial tubule was developed, allowing confocal imaging and the study of the tubular morphology of renal proximal tubule epithelial cells and human umbilical vein endothelial cell (HUVEC) cultures (92).

Earlier, an *in vitro* functional vasculature that mimics the glomerular tuft was reported (93). A glomerulus-on-a-chip could not be developed until functional human podocytes, the cells that regulate selective permeability in the glomerulus, became available. Once a protocol for differentiating the hPSCs into podocytes expressing mature phenotype markers (nephrin^+^, WT1^+^, podocin^+^, and Pax2^−^) and capable of exhibiting primary and secondary foot processes was presented, these podocytes were co-cultured with human glomerular ECs in an OoC microfluidic device (71, 94). In these studies, the function of the kidney glomerular capillary wall was restored on a microfluidic chip. Subsequently, a model for efficient drug screening and nephrotoxicity test using a co-culture of renal proximal tubule epithelial cells and peritubular capillary ECs in a microfluidic kidney chip was proposed (95).

All the models described above can be called renal tissue models. A kidney organoid-on-a-chip, which represents an entire kidney, was described in a study that used a millifluidic device (80). A hiPSC-derived kidney organoid embedded in a gelatin-fibrin (gelbrin) ECM in a 3D-printed millifluidic chip was superfused (flowed around their top surface), which increased the number of EC precursors and then created a vascular network with perfused lumens surrounded by smooth muscle cells and pericytes. This increased the vascularization of the kidney organoid, which will be discussed below.

## 3 Vascularization of kidney organoids

Vascularization of the fetal kidney involves two distinct processes: vasculogenesis and angiogenesis (96, 97). Vasculogenesis is the development of the vasculature through the differentiation of mesoderm-derived angioblasts, the progenitors of ECs. Angiogenesis is the formation of blood vessels from the existing vasculature through sprouting and intussusception (97–99).

During mouse embryogenesis at the stage of UB invasion into the MM (at E11.5 in mice), the MM does not contain a vasculature. The first blood vessels sprout from the dorsal aorta and the common iliac artery. Over the next 24 h, a rich capillary network develops, which then vascularizes the glomeruli. The ECs migrate into the vascular cleft and form single-capillary stage glomeruli (44, 100, 101). At this stage, all of the artery stems unite and form the renal artery at E15.5 (102). This is the general description of the angiogenic model of kidney vascularization. This model was supported by transplantation studies showing that, when the embryonic kidneys were transplanted into murine hosts, the larger vasculature was host-derived (103–106). Recent detailed spatiotemporal analysis of kidney vascularization has shown that the endothelial plexuses in each step of development can always be traced back to the renal artery (107), thus supporting an angiogenesis-only mechanism.

In other studies, it was shown that the progenitors of ECs contribute to the formation of nephron capillaries (28, 108), suggesting that vasculogenesis also participates in the vascularization of the kidney. The presence of *Flk1*, a receptor tyrosine kinase for VEGF and the marker for endothelial progenitors, i.e. angioblasts, in E10 transgenic mice and the transcripts for receptor tyrosine kinases, which are markers of the endothelial precursors (*VEGFR-1*/*Flt-1*, *VEGFR-2*/*Flk-1*, and *Tie-1*) in E11 mouse MM, also support the idea that vasculogenesis takes part in the development of the renal vasculature along with angiogenesis (109, 110). Another study showed that *Foxd1*-positive mesenchymal cells gave rise to a portion of the peritubular capillary endothelium, but not of the glomerular or large vessel endothelium (111). A number of studies where embryonic kidneys were transplanted into murine hosts showed that part of the glomerular vasculature was graft-derived (103, 110, 112). The presence of the endogenous kidney endothelial progenitor cells CD146^+^CD31^−^ derived from the *FOXD1* stroma population was also demonstrated at the E11.5 stage, when the MM from the mouse embryonic kidneys were dissociated, cell sorted, and reaggregated into a kidney organoid that was capable of revascularization (113).

Thus, there are still two points of view on what processes are involved in the formation of the renal vasculature: only angiogenesis and both angiogenesis and vasculogenesis. As it appears, it will be difficult to determine which of these points of view is correct using only *in vivo* data. An *in vitro* model system representing a developing kidney connected to a perfusable vasculature might be a good option to reveal the cellular and molecular basics of kidney vascularization. The more advanced stages of kidney vascularization and maturation of the glomerular vasculature require the migration of various cell types, including mesangial cells, pericytes, and vasculature smooth muscle cells from different embryonic tissues (114–117).

The main purpose of the vascularization of renal organoids is to make the renal organoid functional so that it can perform one of the main functions of the kidney—filtering the blood. Another very important goal is to accelerate the maturation of kidney organoids, which, without blood circulation, will always remain immature. Nutrition and oxygen supply will also help increase the size and the stage of maturity. The perfused vasculature provides paracrine support for the differentiation and maturation of the organ-specific cell types during embryonic kidney development, which is lacking during organoid development without a vasculature. All protocols for the differentiation of PSCs into kidney organoids yield organoids with avascular glomeruli. Two strategies for the vascularization of kidney organoids can be distinguished: *in vivo* and *in vitro*. Both have different advantages and disadvantages. A list of the main protocols for organoid kidney vascularization using hPSCs is presented in **Table 2**.

**Table 2:** Main protocols for human pluripotent stem cell (hPSC)-derived kidney organoid vascularization.

Vascularization protocol	Vascularization type	Reference
Vascularization *in vivo*		
Xinaris et al., 2012 Sharmin et al., 2016 van den Berg et al., 2018 Tanigawa et al., 2018 Bantounas et al., 2018 Tran et al., 2019 Nam et al., 2019 Gupta et al., 2020 Tsujimoto et al., 2020 Takebe, 2015 Francipane et al., 2019 Garreta et al., 2019 Kaisto et al., 2020	Transplantation under the kidney capsule of murine hosts Transplantation into a cranial window of murine hosts Transplantation into a lymph node Transplantation on CAM	(118) (10) (79) (11) (119) (120) (121) (122) (70) (123) (124) (27) (125)
Tarnick et al., 2022		(126)
Vascularization *in vitro*		
Sekiya et al., 2019 Homan et al., 2019 Lee et al., 2021 Menendez et al., 2022		(127) (80) (128) (129)

CAM, chorioallantoic membrane.

### 3.1 Vascularization of kidney organoids *in vivo*

Currently, two methods of *in vivo* vascularization are used: the implantation of organoids into an immunodeficient host animal and the grafting on the chorioallantoic membrane (CAM) of a chicken embryo.

#### 3.1.1 Implantation of kidney organoids into immunodeficient mice

The method of implanting renal organoids under the kidney capsule of immunodeficient mice has been applied by many research groups (10, 11, 70, 79, 118–122).

In one of the first studies on the *in vivo* vascularization of hPSC-derived NP cells, it was shown that the human glomeruli vascularized with the host mouse ECs (10). The hPSC-derived podocytes with nephrin labeled by green fluorescent protein (GFP) were concentrated around the fenestrated ECs. The authors detected evidence of an enhanced maturation of the hPSC-derived glomeruli through the formation of foot processes, slit diaphragm-like structures, a double-layered GBM, and fenestrated ECs.

In a later study, the hPSC-derived kidney organoids were transplanted under the kidney capsule of immunodeficient mice, which also resulted in host-derived vascularization, and it was shown that the nephrons in the organoid were developed to a functional stage (79). The method using live *in vivo* imaging through an abdominal window allows observing the vascularization and functional perfusion of the hPSC-derived kidney organoids using a combination of mouse- and human-derived blood vessels. The structure of the glomeruli was analyzed using transmission electron microscopy, which showed evidence of their enhanced maturation as trilaminar GBM podocyte foot processes and a fenestrated endothelium.

In a later study (119), the hPSC-derived kidney progenitors were dissociated, subcutaneously injected into immunodeficient mice, and formed a fused host- and organoid-derived vasculature in matured glomeruli.

In a recent study (124), the mouse lymph node was used as an alternative transplantation site where the MM can differentiate into mature renal structures with excretory, homeostatic, and endocrine functions and develop a host-derived vasculature.

Subsequently, various methods for the vascularization of kidney organoids *in vivo* were carried out.

A method using the asynchronous mixing of cells differentiated at different times into the same organoid (called the kidney heterochronic organoid) has been shown to stimulate nephrogenesis, with the kidney heterochronic organoids shown to be well vascularized when engrafted under the mouse kidney capsule (122). The connection of the engrafted kidney heterochronic organoid to the systemic circulation by 2 weeks after engraftment was proven using microcomputed tomography and the injection of a circulating vascular marker.

Recapitulation of the nephrogenic niches from separately induced metanephric NP- and UB-like cells, which are subsequently differentiated into glomeruli, renal tubules, and CDs *in vitro*, was succeeded by the use of the selective differentiation protocol for hPSCs. Transplantation under the mouse kidney capsule resulted in the vascularization of the organoids by the host ECs, which was detected using anti-mouse CD31 staining (70). The host ECs integrated into human organoids and vascularized the glomeruli-like structures, as determined by the development of the Bowman’s capsule. To prove that the vascularized network was functional, the transplanted kidney organoids, 10 days after transplantation, were observed with intravital imaging using multiphoton microscopy. In blood vessels migrating into the hiPSC-derived glomeruli, lumens were detected after tail vein injections of rhodamine B-conjugated dextran. Examination of the epithelial connections in the organoids suggested that the hiPSC-derived single NP and UB cells developed vascularized kidney structures with interconnections between the renal tubules and CDs *in vivo*.

Thus, it can be summarized that all hPSC-derived kidney organoids showed higher maturation after implantation into mice, which included the increased expression of the maturation markers, functionality, structural organization and also, in some cases, the formation of a fenestrated glomerular endothelium (79). The survival and further development of kidney organoids *in vivo* was significantly longer (up to 8 months) compared to that in *in vitro* conditions, where the level of vascularization no longer increased after 40 days.

Vascularization *in vivo* probably will not have commercial and clinical applications. Therefore, *in vitro* vascularization should be studied more efficiently (130).

#### 3.1.2 Grafting of mammalian kidney organoids on chicken CAM

Chicken CAM models have a long history in angiogenesis and tumor biology research due to their many benefits. Primarily, the chicken embryo does not have immune reactions to the transplantation of foreign cells and tissues. The immune system develops along with the development of the embryo. Thus, it is not active at least until the late stages of embryo formation, when transplantation experiments are not allowed due to ethical reasons. In addition, the chicken embryo culture is a very convenient model system for microscopic imaging, especially when set up as an *ex ovo* culture (125, 131–136). In kidney research, xenotransplantation to the CAM of avian embryos was used as an intermediate method between cultivating embryonic kidneys *in vitro* and transplanting *in vivo*. In later studies, grafting on chick CAM was used as an approach to provide a vascular and soft *in vivo* environment to kidney organoids (27, 137). CAM has been found to be a suitable microenvironmental niche to support the differentiation and maturation of renal microstructures, such as the kidney organoids and renal microtissue derived from the co-culture of renal progenitors with ECs and mesenchymal stem cells. Characterization of the specific mechanical properties of the CAM and the design of soft synthetic hydrogels that resemble the stiffness of the CAM microenvironment provided a method to enhance the maturation of kidney organoids cultured *in vitro* (112, 137–139).

Previously, it was shown that the mouse kidney primordia are vascularized predominantly by host-derived ECs on CAM (140, 141). The avian endothelium origin of the renal vasculature in the xenotransplanted tissue (140, 141) makes this approach less applicable because the avian–mammalian chimeric embryonic kidney model is more complex for the study of renal vasculature development.

The model system in which the renal vasculature develops from intrinsic ECs and chicken embryo just supplies the blood to the renal organoid has been published recently (27). In this case, analysis of the cell–cell interactions between the podocytes and ECs during glomerular formation will be easier for interpretation.

The hiPSC-derived kidney organoids implanted into CAM exhibited glomeruli with an enlarged Bowman’s space and tubule-like structures with enlarged lumens after 5 days of implantation. Chick CAM blood vessels were found in close vicinity to the glomerulus structures within the implanted organoids. The soft *in vivo* microenvironment of the chick CAM promoted the growth and penetration of the host-derived vasculature to the renal organoid. Hydrogels replicating the same stiffness as that in the *in ovo* CAM microenvironment have been produced, and it was shown that these biomaterials facilitate the differentiation of hiPSC-derived kidney organoids.

Optimization of the renal organoid and organotypic culture on avian CAM for vascularization, extended development, and improved microscopy imaging has been presented recently (125). Combined with the previously published fixed Z-direction method, the new protocol describes how to provide optimal conditions for the long-term confocal imaging of renal organoids and organotypic cultures. Together, these two methods allow enhancing the vascularization and providing the blood flow to the renal organoids and embryonic kidney cultures. In this work, vascularization of the glomeruli by endogenous ECs was demonstrated. This is a crucial difference of CAM grafting *versus* organotypic culture of the embryonic kidney/renal organoid. In the case of *in vitro* organotypic cultivation, ECs never invade the forming glomeruli, which remain avascular. Thus, it can be proposed that the blood flow or even the mechanical forces generated by it are extremely important for the appropriate interactions between ECs and the forming nephrons.

### 3.2 Vascularization of kidney organoids *in vitro*

All protocols for the differentiation of PSCs into kidney organoids have so far failed to generate functional vasculature. Some protocols have resulted in the generation of PSC-derived kidney organoids with immature ECs that do not form blood vessels or migrate into the glomeruli to form a glomerular tuft (6, 21, 79). Various optimizations of these differentiation protocols, such as the addition of VEGF, only increased the number of ECs that are still unable to initiate invasion into the glomeruli (7).

Takebe et al. (123) presented a method for organ bud formation from different tissues by combining PSC-derived tissue-specific progenitors or relevant tissue samples with ECs and mesenchymal stem cells. They showed that, as well as in the case of kidneys, the mesenchymal stem cells initiated condensation *in vitro* within these heterotypic cell mixtures, and these kidney buds, after cranial transplantation into immunodeficient mice, revascularized and were able to efficiently self-organize into functional tissues. Mesenchymal-derived cell-driven condensation *in vitro* with the presence of ECs in the primary mixture of cells demonstrated the ability to self-assemble *in vitro* from the desired multiple cell/tissue types into a complex tissue. This can be regarded as a step forward in the creation of an *in vitro* model of a functional vascularized kidney.

Another study proposed a method to reconstitute both the kidney and its vascular architecture *in vitro* using dissociated and sorted mouse embryonic kidney cells and incorporating ECs into the system and subsequent transplantation (142). This allowed demonstrating that ECs of donor origin make a significant contribution to the formation of arterioles and glomerular capillaries formed after transplantation.

The incorporation of ECs and mesenchymal cells later in a 3D co-culture system with hiPSC-derived kidney precursors and the subsequent vascularization on chicken CAM increased the maturation of renal structures. This demonstrated the possibility of optimizing the *in vitro* step before inducing vascularization by transplantation, grafting on CAM, or using other approaches (137).

Another potentially very effective strategy is to vascularize the kidney organoids entirely *in vitro*. The ability to successfully vascularize the kidney organoids *in vitro* will pave the way for their use in many areas: drug screening, kidney disease modeling, and transplantation, among others. Most importantly, this might elucidate the fundamental mechanism of glomerular vascularization and kidney development in general.

Various attempts have been made to vascularize the kidney *in vitro* (80, 127–129). Filtration of the blood and the production of urine meant that the kidneys are always exposed to the blood and urine flow. It is therefore not surprising that static *in vitro* culture conditions do not result in the proper development of kidney organoids. Therefore, an air–liquid interface perfusion system was developed for the organotypic culture of renal organoids derived from hPSCs (127). This system results in the accelerated organization of epithelial cells and tissues in renal organoids *in vitro*, but not of ECs.

The first promising system was developed as a millifluidic device in which hPSC-derived kidney organoids were cultured under flow (80). The flow conditions and supportive ECM allowed accelerating the maturation of the organoid vasculature. Even the flow along the top surface of the organoids, rather than through it, led to an increase in the number of EC precursors, subsequently allowing them to organize into vessels. The EC vascular structure in the kidney organoids was improved under flow conditions compared to static conditions. Vascular invasion of the glomeruli, organization of the capillary loops, and partial perfusion through the lumen were observed. An important condition was the absence of exogenous VEGF. Only flow particularly enhances the development of the glomerular vasculature.

A similar kidney organoid-on-a-chip system providing fluid flow that mimics shear stress with optimized ECM conditions (Matrigel with VEGF) also demonstrated that kidney organoids cultured in fluid flow conditions exhibit more mature podocytes and vascular structures compared to the static culture condition (128).

A later published study (28) showed that optimizing the differentiation protocol for hiPSC-derived kidney organoids through the precise modulation of Wnt signaling led to the establishment of a specific glomerulus-to-tubule ratio. Thus, the appropriate amount of VEGF was obtained, which coordinated the development of a larger volume of ECs and a higher level of development of the resident vascular network. Functional validation based on the dextran uptake showed that the vasculature developed *in vitro* correlated with a similar dextran uptake assay performed using kidney aggregates derived from E12.5 mouse embryonic kidneys. In summary, this study successfully induced the vascularization of organoids *in vitro*, but further development of the blood perfusion system is still needed.

Recently, another important step has been made toward the *in vitro* vascularization of renal organoids (129). A novel OoC system supports the culture of kidney organoids that enhances the maturation of endothelial populations based on co-localization analysis of the endothelial markers. In addition, the migration and proliferation of HUVECs cultured in the channels of the chip within the organoid tissue was observed. An interconnection has been found between the HUVECs and endogenous ECs. Later, structures were formed that represent hollow endothelial tubules, reminiscent of blood vessels. As it appears, this work accomplished the closest match to the requirement of the development of an *in vitro* vascularized kidney model with the perfusion organized through endothelial vessels. What is still lacking in this approach is a demonstration of the glomerular vascularization. In our point of view, the formation of the glomerular tuft is the most important hallmark of the development of a functional kidney. Thus, it could be an important goal of future research.

### 3.3 ECs of the kidney blood vascular system and molecular mechanisms of glomerular vascularization

The mammalian vascular system consists of the blood and the lymphatic vascular systems. Blood vessels are lined with blood ECs, and lymphatic vessels are correspondingly lined with lymphatic ECs. Both types of ECs have their own unique structure and gene expression profiles based on specific markers identified for them (143). Blood vessels are composed of ECs and vascular mural cells (VMCs), including pericytes lining the microvessels and vascular smooth muscle cells lining the large diameter vessels such as arteries and veins (144, 145). ECs line the inside of the tube and are in contact with the flowing blood, while vascular mural cells control the vascular tone and provide structural support (146).

**Figure 2:**
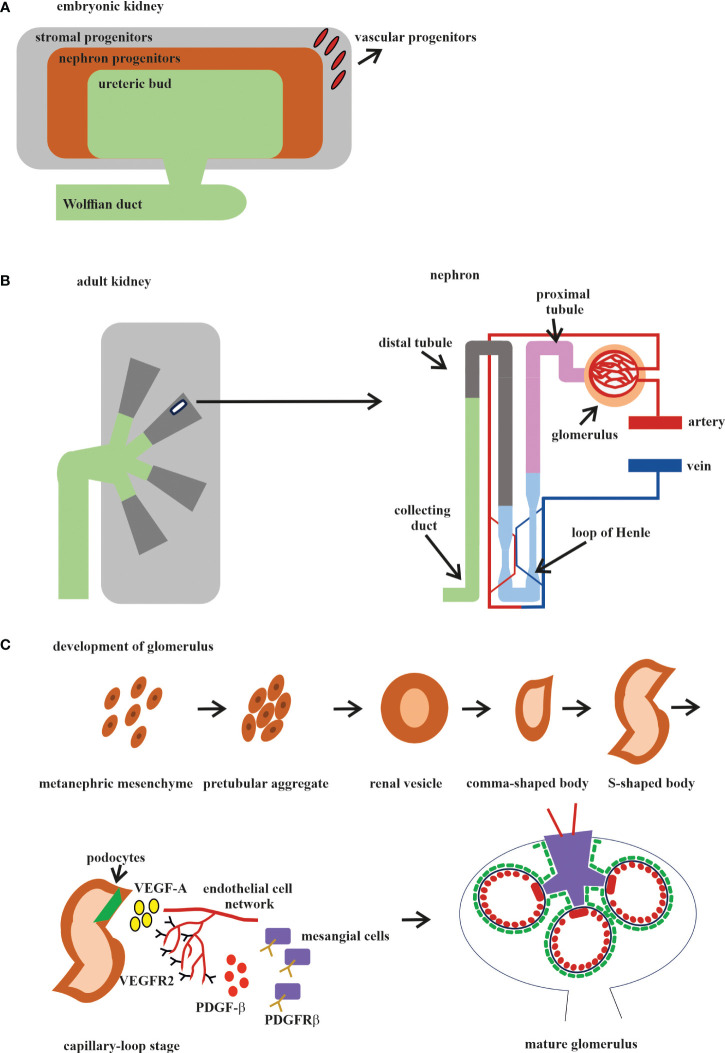
Development of the human kidney. **(A)** Embryonic kidney. There are four precursors of the embryonic kidney: stromal progenitors (*SP*, marked in *gray*), nephron progenitors (*NP*, marked in *brown*), ureteric bud (*UB*, marked in *green*), and vascular progenitors (*VP*, marked in *red*). **(B)** Adult kidney. The renal pelvis and ureter (marked in *green*) originate from the UB. Simplified schematic overview of the nephron and glomerular vasculature. Blood filtration occurs in the glomerulus (marked in *beige*). The filtered blood is transported to the proximal tubules (marked in *pink*), loop of Henle (marked in *blue*), and distal tubules (marked in *gray*) and exits through the collecting ducts (marked in *mustard*). **(C)** The development of the glomerular consists of five stages: *1*) vesicle; *2*) comma-shaped body; *3*) S-shaped body; *4*) glomerular capillary loop stage; and *5*) mature glomerulus. At the capillary loop stage, the podocyte precursors (marked in *green*) release vascular endothelial growth factor A (VEGF-A; marked in *yellow*) to recruit endothelial cells (ECs) in the kidney mesenchyme, which contains VEGFR2. ECs are transferred to the vascular cleft, where they continue their differentiation in close proximity to VEGF-A-producing podocytes. Glomerular ECs express platelet-derived growth factor beta (PDGF-β; marked in *red*) and recruit PDGF receptor beta (PDGFR-β)-containing mesangial cells (marked in *violet*) into the developing glomerular tuft.

Different organs perform different functions, and this is why ECs from different organs acquire their own specialization and specific gene expression profile during development. The kidney has very specialized functions and has a blood vascular system that is different from those of other organs (143). In the embryonic kidney, the VPs, SPs, and NPs are derived from the MM. VPs and SPs co-localize at the E11.5 stage, as it was reported that the endothelial progenitors CD146^+^CD31^−^ are derived from the *FOXD1* stroma population (113) (**Figure 2A**). Both the VPs and the invaded vasculature form the endothelium system of the nephron in adult kidney (**Figure 2B**). All three types of cells that form blood vessels—ECs, pericytes, and vascular smooth muscle cells—are closely associated with the renal epithelial system (146) (**Figure 2B**). The ECs of the renal vasculature have a very high level of heterogeneity, depending on which part of the kidney they are found in and, accordingly, what function that part of the kidney performs (143) (**Figure 2B**).

Both the endothelia and VMC progenitors are present in the MM and differentiated by the *FOXD1* gene at the mE10.5 stage (44, 147). They vascularize the glomerulus through the synergistic processes of vasculogenesis and angiogenesis, which were described above. All of the major stages of kidney vasculature development occur at mE10.5–mE15. Analysis of the single-cell transcriptome of hPSC-derived organoids generated using two different protocols and compared with human kidneys at 16 weeks and with adult kidneys showed that the organoids contained 12 distinct types of kidney cells, but their level of maturation was closer to that of fetal kidney cells than to adult kidney cells (74). The spatiotemporal heterogeneity and the patterning of the developing kidney blood vessels have been studied to understand when and how endothelial regionalization occurs (148). An in-depth anatomical and molecular analysis of the developing kidney endothelium confirmed that ECs grow synchronously with the UB already at stage mE10.5 and then begin to show signs of specification at stage mE13.5, when the first arteries can be identified. Transcriptional profiling has identified a long list of novel genes enriched in kidney ECs, expressed in regionally defined and organ-specific patterns, and has shown that the renal endothelium exhibits marked heterogeneity as early as mE15.5 and that many of the EC gene expression patterns are highly dynamic over time. The association of ECs with UB was analyzed by immunofluorescence (IF), which showed that, from stage mE14.5, this association started and was maintained for the rest of the embryonic kidney development, except for the renal pelvis, where the ECs were closely associated with the epithelial cells of the renal papilla, but not with those that constitute the ureter. The next step forward in the study of the progression of the molecular pathways that determine kidney vascular heterogeneity has been the study using bulk and single-cell RNA-seq of the kidney vasculature at different stages of development, which revealed distinct transcriptomic signatures associated with the different kidney vasculatures (149). In this study, several genes coding transcription factors and their putative targets that define the ultrastructural, phenotypic, operational, and functional identity of the glomerular capillaries and the surrounding vascular network were identified. The transcription factor *Tbx3*, which is robustly expressed in glomerular ECs and regulates the transcriptional network that determines the specification and function of glomerular ECs, has been characterized. This work traced the development and specialization of the renal vasculature, starting from the mE14 stage, in the process of maturation into adult vessels. The period between mE10.5 and mE14 was not included in this work. It should also be considered that, in this work, only the EСs were analyzed and that the influence of other cell types was not taken into account. Current knowledge of the phenotypic and molecular heterogeneity of the kidney endothelium provides a very detailed list of the expression markers specific to each defined subpopulation of renal ECs (143).

The glomerulus, the filtering unit of the kidney, consists of four types of cells: the fenestrated ECs, the epithelial cell podocytes, the perivascular mesangial cells, and the parietal epithelial cells (150, 151). The podocytes, GBM, and the fenestrated ECs form the blood-filtering structure called the glomerular capillary tuft. Mesangial cells provide the rigidity and integrity of the glomerular tuft and serve as its structural framework. Parietal epithelial cells form the Bowman’s capsule surrounding the glomerular tuft. Glomerular development is generally described in five steps: the renal vesicle, the comma-shaped body, the S-shaped body, the glomerular capillary loop stage, and the mature glomerulus (**Figure 2C**).

Glomerulogenesis consists of one step before the invasion of ECs (the vesicle stage), then is divided into two steps (the comma-shaped body and the S-shaped body) when the podocyte progenitors begin to express VEGF and the MM-derived glomerular EC precursors expressing *Foxd1*, *VEGFR2* (*FLk1*/*Kdr*), and *VEGFR1* (FLT) begin to interact with the podocytes. During the last two steps (the glomerular capillary loop stage and the mature glomerulus), all four cell types interact with each other and the mature glomerulus (147, 150, 152) (**Figure 2C**). Among the growth factors, VEGF is a fundamental regulator of EC differentiation, migration, and cell–cell interactions, including the sprouting angiogenesis stimulation and tip cell activation (153). VEGF pathway interactions initiate the migration of ECs into the vascular cleft of an S-shaped glomerulus. The Notch pathway regulates the expression of all the VEGF receptors (VEGFRs). At the same time, *VEGFA* directly stimulates the Notch pathway during vascular development. Subsequent signaling through *VEGFR2* requires the presence of VEGFR3, ephrin-B2, neuropilin-1, the thrombospondin receptor CD47, VE-cadherin, and some integrins (154, 155).

In the first phase, the glomerular EC precursors form a capillary chord without a lumen; subsequently, due to the expression of TGF-β by podocyte precursors, the formation of a capillary lumen occurs. This process requires VEGF to induce apoptosis, through which excess ECs will be eliminated (156, 157). The ECs of the glomerulus at the late stage of the S-shaped body form the fenestrated endothelium along with foot process formation in overlying podocytes (**Figure 2C**).

Glomerular ECs expressing platelet-derived growth factor beta (PDGF-β) attract PDGF receptor beta (PDGFR-β)-expressing mesangial cells, which migrate into the cleft and participate in glomerular maturation (150).

The chemoattractant cytokine C-X-C chemokine ligand 12 (CXCL12) expressed in podocytes during the S-shaped body stage requires its receptor C-X-C chemokine ligand 4 (CXCR4) expressed in ECs, suggesting an intimate crosstalk between these cells by paracrine signaling.

Simultaneous with the formation of the capillary chord in the glomerulus, the angiopoietin–Tie signaling axis determines the formation of a vessel sprouting from remnant glomerular EC precursors. The mesenchymal cells surrounding the developing vasculature express Ang1, which promotes vessel stability and inhibits fibrosis in a paracrine manner (158). ECs are the main Ang2-expressing cells that have an autocrine function and participate in context-dependent regulation. The angiopoietin receptors Tie1 and Tie2 are expressed predominantly in ECs. Following the invasion of ECs to form glomeruli, podocytes and ECs undergo synergistic differentiation to create a filtration barrier.

Immediately after the appearance of blood flow in the capillary system, the luminal surface of the endothelium begins to be constantly exposed to and respond to shear forces. Vascular development occurs through a series of flow-sensitive processes. Shear stress regulates multiple processes in vascular development, including sprouting, EC apoptosis and migration, and arterial fate specification (159). Numerous transcriptome studies of ECs under different flow conditions have shown that shear stress regulates the signaling pathways BMP/TGF-β, Hippo/YAP/TAZ, Notch, Wnt, and HOX (160). All of these pathways participate in kidney nephron development and in endothelial vasculature formation and maturation.

The formation of blood vessels is the result of the interaction of perivascular cells and the extracellular matrix, which has viscoelastic properties and exposes the cells to traction stress (161). The transition from a 2D to a 3D culture using different matrices in the context of organoids not only better resembles the *in vivo* situation but also ensures the correct cell fate: when using the same differentiation protocol, the initial 2D culture of undifferentiated PSCs has been shown to result into differentiation into cardiac lineages, whereas the initial 3D culture directs differentiation toward the kidney (6). The choice of a 3D matrix for renal organoid culture is critical because the composition of the ECM also influences cell fate (162). Currently, several types of ECM have been developed for 3D culture, including animal-derived ECM and synthetic matrices (163). The ECM obtained from animal tissues (Matrigel) was, for a long time, the only published matrix that allows the expansion, differentiation, and passage of renal organoids (163). However, well-known disadvantages of Matrigel, such as the batch-to-batch variability and the presence of growth factors, make complete control of the ECM impossible. In addition, Matrigel does not reflect the organotypic ECM of the kidney and is not suitable for transplantation studies. Despite this, Matrigel with VEGF has been used to vascularize the kidney organoid-on-a-chip in several reports (128). Tissue-specific types of ECMs isolated through decellularization and digestion of the kidney have also been tested for the optimization of the protocols of renal organoid generation (164, 165). Hydrogels made from natural materials, including gels based on collagen, fibrin, and polyglycans, are most often used for the fabrication of kidney-on-a-chip (166). Gelbrin (80) or fibrin-based hydrogels have been reported to show the best results in supporting renal organoid formation and in *in vitro* vascularization (129).

### 3.4 Different possible strategies for targeted vascularization of a kidney organoid-on-a-chip

**Figure 3:**
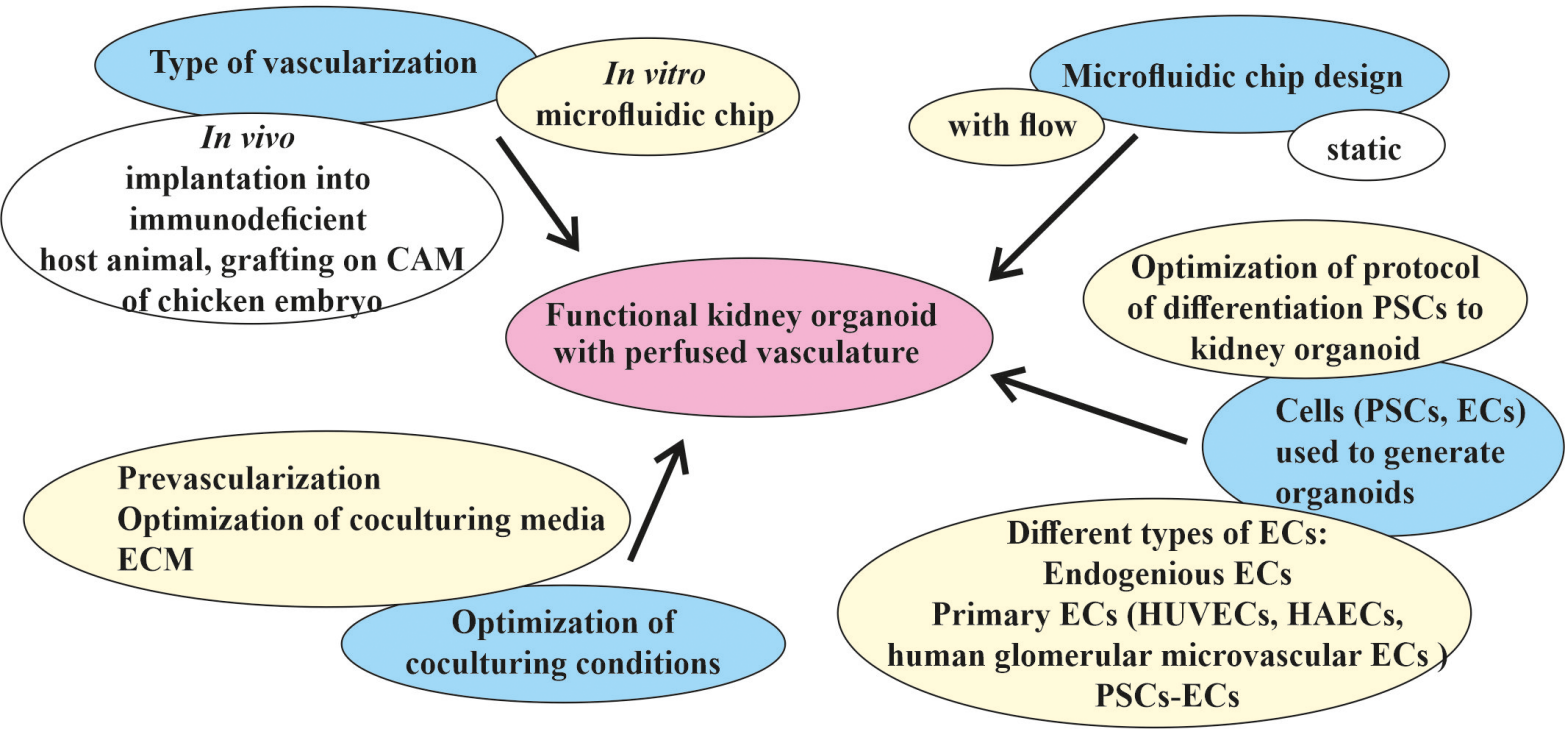
Different strategies for the targeted vascularization of kidney organoids *in vitro* in a microfluidic chip. The major goal of generating a functional kidney organoid with perfused vasculature *in vitro* (indicated in *pink*) has not yet been achieved. The main efforts (marked in *blue*) were spent in the development of *in vivo* and *in vitro* methods for the vascularization and engineering of different types of microfluidic devices adopted for the cultivation of kidney organoids *in vitro* and the selection of protocols of differentiation, types of cells, and the culture conditions for the generation of organoids. Two types of vascularization were implemented in an *in vivo* format: implantation into an immunodeficient host animal and grafting into a chicken embryo chorioallantoic membrane (CAM). One type of *in vitro* kidney organoid vascularization in a microfluidic chip format potentially leads to the generation of a functional kidney organoid with a perfusable vasculature. The most promising microfluidic chip design is the flow-enabled type. The main source of kidney organoids and vasculature are pluripotent stem cells (PSCs), which can be differentiated using different protocols and different types of resulting cells. Optimization of the co-culture conditions includes pre-vascularization, medium, and extracellular matrix (ECM) optimization for co-culture. The most promising strategies for the *in vitro* vascularization of kidney organoids in microfluidic chips discussed in this review are indicated in *yellow*.

The discovery of better treatments for kidney diseases requires developing improved model systems to study the disease mechanisms and examining new therapeutic approaches. A functional *in vitro* vascularized kidney organoid from patient-derived hPSCs could open new opportunities in the field of regenerative kidney medicine. Depending on how the problems that hinder the achievement of this goal were identified, different strategies must be chosen (**Figure 3**).

One direction is to optimize the protocol of the differentiation of PSCs into a kidney organoid. More precise tuning of Wnt signaling during kidney organoid differentiation has provided a way to produce the glomeruli and tubules in a specific ratio, allowing podocytes to produce the appropriate amount of VEGF to attract EC precursors, leading to their more proper development and infiltration into the glomeruli (28). It is unclear whether this ratio corresponds to that of the glomeruli and tubules in the embryonic kidney, but it does result in a higher level of development of the resident vasculature in the organoid. Any future improvement in the differentiation protocol, whether it follows a natural process or not, would be beneficial. In general, the method of following the natural process leads to better results, but requires a more detailed study of the embryonic kidney, which is made possible by modern methods.

Optimization of the differentiation protocol also includes the use of exogenous ECs, such as primary ECs (i.e., HUVECs and human glomerular microvascular ECs) (80). In this particular protocol, exogenous cells did not appear to improve vascularity; however, potentially using a different EC type increases the opportunity to determine an effective way of optimizing the protocol. The generation of other non-renal organoids (e.g., cardiac spheroids) involves the step of adding hPSC-derived ECs, which appears to be a potentially very promising method of improving the maturation of other cell types, not just ECs (167). An attempt to co-differentiate the hiPSCs into the renal and endothelial lineage has been made previously (6, 21–23, 28). Some of the important factors in vascularization in engineered tissues include the type of ECs used and their ratio to PSCs or other cell types (143, 168). The recently published first PSC-derived vascular organoid model consisting of ECs and pericytes has shown significant lumen formation (169, 170). Given the common mesodermal origin of the kidney and ECs, attempts at co-differentiation or co-cultivation within the organoid seem quite logical. The ability of modern methods to perform assessment of the cellular heterogeneity and enrichment of the different populations also appears to be a very promising strategy for optimizing the kidney organoid differentiation protocol.

It has been convincingly shown that, between static and flow conditions, the latter significantly increases the level of maturation of the kidney organoids (80). However, the manner in which this flow passes can be designed in different ways (129). The development of new microfluidic or other engineered solutions could be a very promising strategy to enhance the influence of flow on kidney organoid maturation.

The search for the most suitable ECM is ongoing (80, 128, 139). Influencing cell fate using the correct ECM could offer additional ways to optimize the kidney-on-a-chip model.

Various strategies from the organoid field have been used to create a vascularized kidney organoid-on-a-chip, and others should also be applied. The methodological challenge of co-culturing kidney organoids and ECs forming the vasculature lies in the different media required by these two organs. In a generalized method for organ bud formation from diverse tissues through combining embryonic kidney-derived dissociated cells with ECs and mesenchymal stem cells, a medium consisting of an EC medium and a kidney cell medium in a 1:1 ratio was used (123). In a more recent paper on enhancing the maturity of the renal progenitors in renal microtissue derived from the co-culture of renal precursors with endothelial and mesenchymal stem cells, the ratio of the three cell types was optimized, but the ratio of the two different media was the same as that in the above-mentioned article (137). Another existing strategy for generating various organoids is pre-vascularization (171). This allows first using a specific EC medium to create a perfused vasculature and then moving on to an organ-specific medium. Using only one type of medium for both the development of an organoid and the creation of a perfused vascular network seems impractical. A new method of culturing 3D tissue, i.e., placing it directly on a “vascular bed-on-a-chip,” could potentially be applied to a kidney-on-a-chip model as well (172).

## 4 Application of kidney organoids in biomedical research

The emergence of methods aimed at differentiating human PSCs into kidney organoids has intensified the study of the basic mechanisms of embryonic kidney development. A more detailed understanding of these mechanisms and the ability to manipulate factors that determine cell fate have led to various possible applications of this knowledge for the treatment of kidney diseases. The ability to obtain kidney organoids from patient cells has opened enormous opportunities for the study of human kidney development and diseases.

The ability of organoids to restore the features of a complex disease, such as polycystic kidney disease (PKD), has been exploited to create a reproducible, universal 3D framework to model human epithelial diseases (5–7). The discovery that the knockout of the PKD genes *pkd1* or *pkd2* induces the formation of renal tubular cysts in hPSC-derived kidney organoids has created an innovative human organoid model for kidney injury and disease (6). By combining the PKD organoids with the systematic replacement of the physical components of the environment, a human cellular system that simulates PKD with high efficiency and specificity was created (5). Comparison of the PKD and non-PKD organoids demonstrated the special role of the microenvironment and adhesion in the early stages of the disease. A test for the nephrotoxicity of cisplatin and forskolin using gene-edited kidney organoids extended this analysis to specific biomarkers and showed that the myosin pathway is involved in PKD (7). The development of an *in vitro* method for culturing kidney organoids under flow on millifluidic chips has made it possible to uncover the pathology of the disease and to develop treatments for autosomal-recessive PKD. Transcriptomic analysis identified the mechanosensitive molecules *RAC1* and *FOS* as potential therapeutic targets, which was confirmed in patient kidney samples. Based on this discovery, new drugs targeting *RAC1* and *FOS* were assessed in an OoC model under flow conditions that inhibit cyst formation (8).

Drug nephrotoxicity is an important cause of acute kidney injury in critically ill patients (173). A nephrotoxicity test for gentamicin and cisplatin showed that the hPSC-derived kidney organoid system *in vitro* can be used as a patient-specific model of toxic kidney injury (9).

The kidney OoC system designed with fluidic flow mimicking shear stress and Matrigel with VEGF as an ECM demonstrated more matured podocytes and vascular structures and a higher sensitivity to nephrotoxic drugs when compared with the same culture in static conditions (128).

The *in vivo* glomerular tuft vascularization method demonstrated that hPSC-derived organoids can be used as a model to study glomerulonephritis (10). The temporal and spatial expression of VEGF determines its biological activity as a critical player in the regulation of the glomerular filtration barrier. A list of diseases based on glomerular disorders, such as preeclampsia and thrombotic microangiopathies, diabetic nephropathy, HIV-associated nephropathy, crescentic glomerulonephritis, and membranoproliferative glomerulonephritis, might be influenced by the modulation of VEGF signaling (150). Besides VEGF, other signaling pathways such as ANGPT, EGF, SEMA3A, TGF-β, and CXCL12 also communicate between podocytes, the endothelium, and the mesangium within the glomerular capillaries to maintain the filtration barrier function. Glomerular diseases, which are characterized by the dysregulation of these pathways and their stimulation or inhibition, comprise a strategy for discovering new therapeutic solutions (150).

Kidney organoids obtained from the PSCs of patients with hereditary kidney diseases can be used to functionally test new gene variants and to investigate the underlying pathogenetic mechanisms.

Abnormalities of glomerular podocytes can be recapitulated in kidney organoids from iPSCs obtained from a patient with congenital nephrotic syndrome of the Finnish type: mutations in the *NPHS1* gene, which encodes nephrin, lead to the impaired formation of the slit diaphragm in glomerular podocytes. A kidney patient organoid model derived from PSCs showed that slit diaphragm abnormalities occur in podocytes during the early stage of congenital nephrotic disease (11).

Renal tubule-related diseases can also be modeled using kidney organoids derived from the PSCs of patients. Nephronophthisis, which is caused by mutations in intraflagellar transport protein 140, and patient-derived kidney organoids showed short primary cilia and disruption of apicobasal polarity in the kidney tubular epithelium. The correction of this gene through editing rescued these phenotypes (12).

Introducing disease-causing mutations using gene editing technology allows the creation of iPSC-derived human kidney organoids for the study of disease mechanisms. The introduction of mutations in the genes *PKD1* and *PKD2*, which encode the autosomal-dominant PKD-associated proteins polycystin 1 and polycystin 2, respectively, in human iPSC-derived kidney organoids resulted in the formation of cysts (13, 14). The feasibility of using an autosomal-dominant PKD kidney organoid model as a drug screening platform has also been demonstrated (14).

It should be noted that there are a number of challenges that need to be addressed before kidney organoids reach clinical levels. The reproducibility of the differentiation protocols is one of these challenges. The very high level of diversity of the hPSC lines increases the diversity of the resulting organoids, but at the same time does not allow formalization of the protocol (72). The insufficient level of maturity and functionality of renal organoids is a key problem that can only be solved when a reliable method to vascularize organoids *in vitro* is discovered.

## 5 Discussion

The development of protocols for the differentiation of PSCs into kidney organoids opens new opportunities for kidney research to study and treat kidney diseases. Among the first protocols, some have resulted in the differentiation of cells into an organoid to a phase where a population of immature ECs appeared outside the cleft (6, 21–23). The optimization of protocols slightly advanced the differentiation process to the stage where a more mature vasculature was developed (7, 28). *In vivo* vascularization has demonstrated that the development and maturation of the endothelial system is accompanied by the maturation and emergence of nephron functionality. When attempting to vascularize the kidney organoid *in vitro*, the flow conditions and the influence of the ECM were considered (80). To date, there is no *in vitro* glomerular-perfused vascularized kidney organoid model.

Glomerular-perfused vascularization is necessary to promote the development of the capillary network of the glomerular tuft and the maturation of ECs and other organoid cell types and to ensure the supply of nutrients and oxygen in order to achieve a larger organoid size and longer survival. The reasons for vascularization not occurring probably include the lack of proper interaction among all of the cell types that make up the embryonic kidney and the lack of adequate shear stress caused by the flow of a medium that mimics the flow of blood. Technical challenges hindering successful vascularization include the requirement of different media suitable for different cell types, the lack of a suitable EC line for vascularization, and the search for a more suitable ECM.

The methodological challenge of co-culturing kidney organoids and ECs forming the vasculature lies in the different media required by these two organs. One existing strategy for the generation of various organoids is pre-vascularization (171). This allows first using a specific EC medium to create a perfused vasculature and then moving on to an organ-specific medium. Furthermore, it is possible to apply the method of gradually replacing the second medium.

Most attempts to vascularize the kidney organoid with an exogenous EC line have been limited to the use of HUVECs (80, 129), and the poor progress in this field can be attributed to the lack of compatibility between HUVECs and the kidney. In addition, human glomerular microvascular ECs and human aortic ECs were used, but did not show a decisive improvement. Different protocols for the differentiation of PSCs to ECs have been published (174, 175). One of the possible advantages of differentiating the kidney organoid and ECs for its vascularization from the same source of PSCs is that both organs are of mesodermal origin and the first steps of the differentiation protocol should be the same. A detailed comparative analysis of PSC-derived ECs and kidney ECs has not yet been published. The relative distribution of VEGFR2 as critical players in the interaction with the VEGF produced by podocytes could potentially determine a more suitable candidate for the vascularization of the kidney organoid. Moreover, the specific ratios of the different cell types can be fine-tuned depending on the different expression levels of the key molecular players in the signaling pathways of the differentiation protocols. Since the more mature characteristics of the endothelium, such as the venous or arterial phenotypes, will play a role in the next stage of maturation accessible to the *in vitro* system, it is necessary to study the mechanisms by which the maturation process can be controlled. This may partly be the result of the communication with other cell types. The co-culture of PSC-derived ECs with mural cells such as pericytes has been known to result in the formation of human blood vessel organoids (169). This is reminiscent of the behavior of HUVECs with mural cells or a comfortable environment in microfluidic chips *in vitro*. It appears that the same mechanism underlies the phenomenon of mesenchymal stem cells enhancing the vasculogenic and pro-angiogenic activity of endothelial colony-forming cells during engraftment *in vivo* (176). In another study, a similar result was obtained, which showed macrophages enhancing the vascularization of tissue-engineered scaffolds (177). A partly successful result was obtained when attempting to repopulate the glomeruli from a decellularized kidney scaffold using ECs derived from PSCs (178). These studies have accumulated experience in the use of PSC-derived ECs, which can be used for vascularization in microfluidic chip *in vitro*. Adding another cell type to the system might reveal additional mechanisms of tissue/organ formation and help in the production of markers associated with higher levels of maturity.

The search for a suitable ECM is ongoing, mainly in attempts to recapitulate the natural renal ECM (164, 165, 179). However, the main difficulty again lies in the co-cultivation of the endothelial vasculature and the kidney organoid, where each organ requires a specific ECM. Promising results in the maturation of glomerulus-like structures using scaffold materials such as a decellularized renal ECM could potentially be a solution, but only a temporary one (180). In any case, the later large-scale use of vascularized kidney organoids will require a more accessible ECM.

The glomerular tuft consists of two main types of cells—the podocytes and the endothelium—which provide the function of the filtration barrier (150). However, a third type of cells, i.e., the mesangial cells, is indispensable for a mature glomerulus since these cells provide a structural integrity to the filtration unit of the glomerulus. The addition of mesangial cells to kidney organoids and a deeper understanding of their contribution to the glomerular structure and function remain to be accomplished.

The diversity and ratio of the different cell types in the embryonic kidney is different from that in the kidney organoid (68). This is one of the reasons for the insufficient maturation and the lack of functionality of the renal organoids generated so far.

The lack of shear stress determines the incomplete maturation of the endothelial vasculature of an *in vitro* kidney organoid model. The most advanced model uses flow along the top surface of the organoid (80). However, one of the problems is how to design a flow that would pass through the organoid and lead to the luminization of the newly formed capillaries.

One promising prospect is the use of patient-derived PSCs for personalized medicine. NP cells derived from hPSCs from patients undergoing hemodialysis due to disease could be developed into nephron-like structures and used as a source for future applications in tissue engineering (9, 10). The technical issue of selecting and evaluating the correct cells from other tissue cells has not yet been fully resolved.

## 6 Conclusions

The discovery of the methods to differentiate PSCs into organoids represents a major technological breakthrough achieved in recent years. The kidney is a very complex organ in terms of structure and function, the first stages of development of which were reproduced by studying kidney organoids. The mechanism of fetal kidney development can now be studied *in vitro*. Microfluidic devices designed and identified for use in the biomedical field have been applied in combination with kidney organoids, allowing great advances in the field of developmental biology. The successful application of kidney organoids in disease modeling has opened bright perspectives for drug screening and personalized medicine. Nevertheless, despite these great achievements, a functional *in vitro* model of adult or embryonic kidney that is suitable for biomedical research has not been presented yet. One of the major limitations in growing kidney organoids is the lack of a functional vasculature. The endothelial system is one of the main parts of the kidney, the correct development of which stops the further maturation of the kidney organoid as an integral organ. Newly formed capillaries deliver shear stress signals, nutrients, and oxygen to nearby cell populations. The endothelium of the vasculature mediates both paracrine signaling and basement membrane interactions with other cell types, which could potentially improve the maturation of renal organoids. Moreover, the currently unknown causes and mechanisms that remain to be discovered should also need to be considered. The development of cost-effective and large-scale PSC-derived kidney organoids could become a viable option in the nearest future. Further studies are needed to discover a more suitable PSC-derived EC line compatible with the kidney organoid and to learn to manipulate the blood vessel formation and maturation *in vitro*. Tissue engineering technologies such as scaffolds, bioprinting, or organoid-on-a-chip devices will facilitate the development of new kidney organoid models relevant for basic and clinical research, regenerative medicine, pharmacology, and personalized medicine.

## Author contributions

IR: Writing – original draft, Writing – review & editing. MN: Writing – review & editing. YS: Writing – review & editing. SV: Writing – review & editing. IS: Writing – review & editing, Writing – original draft.
